# Characterization of MicroRNA Expression Profiles and the Discovery of Novel MicroRNAs Involved in Cancer during Human Embryonic Development

**DOI:** 10.1371/journal.pone.0069230

**Published:** 2013-08-02

**Authors:** Yi Lin, Yan Zeng, Fan Zhang, Lu Xue, Zan Huang, Wenxin Li, Mingxiong Guo

**Affiliations:** 1 State Key Laboratory of Virology & College of Life Sciences, Wuhan University, Wuhan, China; 2 Department of Biochemistry and Molecular Biology, College of Life Sciences, Wuhan University, Wuhan, China; University of Nevada School of Medicine, United States of America

## Abstract

MicroRNAs (miRNAs), approximately 22-nucleotide non-coding RNA molecules, regulate a variety of pivotal physiological or pathological processes, including embryonic development and tumorigenesis. To obtain comprehensive expression profiles of miRNAs in human embryos, we characterized miRNA expression in weeks 4-6 of human embryonic development using miRNA microarrays and identified 50 human-embryo-specific miRNAs (HES-miRNAs). Furthermore, we selected three non-conserved or primate-specific miRNAs, hsa-miR-638, -720, and -1280, and examined their expression levels in various normal and tumor tissues. The results show that expression of most miRNAs is extremely low during early human embryonic development. In addition, the expression of some non-conserved or primate-specific miRNAs is significantly different between tumor and the corresponding normal tissue samples, suggesting that the miRNAs are closely related to the pathological processes of various tumors. This study presents the first comprehensive overview of miRNA expression during human embryonic development and offers immediate evidence of the relationship between human early embryonic development and tumorigenesis.

## Introduction

MicroRNAs (miRNAs) are ~22-nucleotide (nt) non-coding RNA molecules that regulate gene expression at the level of messenger RNA degradation or translation, exert essential functions in disparate biological processes ranging from cell cycle regulation, differentiation, and metabolism, to normal tissue and embryo development, and even tumorigenesis [1–4]. Alterations in miRNA expression are involved in the initiation, progression, and metastasis of human tumors [5]. Gradually increasing evidence suggests a direct link between miRNAs and many diseases, particularly cancer.

Using various experimental methods, previous studies in animal embryogenesis found that certain miRNAs were continuously reported, and most play crucial roles in differentiation or the maintenance of tissue identity [6–10]. Human development in the first 8 weeks of embryogenesis is potentially one of the most exciting areas of biological research. Specifically, some important tissues and organs, including neurogenesis and the development of the liver, began to form during weeks 4-6 (Carnegie Stages 10-17) of human embryogenesis [11]. Recently, our research group and others have characterized the transcriptional profiles of genome-wide mRNA expression during human embryogenesis using microarray [12,13]. However, human miRNA expression in this period remains to be elucidated.

Many studies have demonstrated that miRNAs are implicated in various aspects of tumorigenesis and animal embryogenesis (Reviewed in 4,5,14–21). In 1892, the French biologists Lobstein and Recamier speculated the concept of the embryonic origin of tumors for the first time. In the 1970s, Dr. Pierce proposed the theory ‘cancer, a developmental biology’ and pointed out that tumorigenesis was intimately involved with developmental biology to a large extent [22–27]. Currently, however, relevant evidence of the relationship between early human embryo development and tumorigenesis is still limited [28–32]. To fill this important gap in knowledge, therefore, more studies are necessary.

In this study, the miRNA expression profiles during human embryonic development were identified and characterized using miRNAs microarrays. The results show that nearly all miRNAs of known function that are highly expressed or exhibit expression changes during human embryonic development have been implicated in several malignant diseases. Furthermore, tissue expression investigations of several miRNAs of unknown function that are highly expressed or undergo expression changes during human embryo development indicate that the expression of these miRNAs was significantly different between cancer samples and corresponding normal tissues, thereby suggesting that these miRNAs may also play important roles in tumorigenesis. These results provide evidence regarding the close relationship between human embryogenesis and carcinogenesis, and further reveal that miRNAs related to embryonic development with unknown functions may be involved in the development and progression of cancer.

## Materials and Methods

### Embryo collection and cell line

Human embryo collection was performed as previously described [13]. Appropriate written consent was obtained from the patients and approval gained from the Medical Ethics Committee of Zhongnan Hospital at Wuhan University by following national guidelines.

### Microarray and Bioinformatics Analyses

The microRNAs microarray assay was performed using a service provider (LC Sciences). The assay started with 2 to 5 µg total RNA sample, which was size fractionated using an YM-100 Microcon centrifugal filter (Millipore). The isolated small RNAs (<300 nt) were 3’-extended with a poly(A) tail using poly(A) polymerase. An oligonucleotide tag was ligated to the poly(A) tail for later fluorescent dye staining; two different tags were used for the two RNA samples in dual-sample experiments. Hybridization was performed overnight on a *μ*Paraflo™ microfluidic chip using a microcirculation pump (Atactic Technologies) [33,34]. On the microfluidic chip, each detection probe consisted of a chemically modified nucleotide-coding segment complementary to target microRNA (from miRBase, http://www.mirbase.org/) or other RNA (control or customer defined sequences) as well as a spacer segment of polyethylene glycol to extend the coding segment away from the substrate. The detection probes were generated from *in situ* synthesis using PGR (photogenerated reagent) chemistry. The hybridization melting temperatures were balanced by chemical modifications of the detection probes. Hybridization used 100 µL 6xSSPE buffer (0.90 M NaCl, 60 mM Na_2_HPO_4_, 6 mM EDTA, pH 6.8) containing 25% formamide at 34° C. After hybridization, detection used fluorescence labeling with tag-specific Cy3 and Cy5 dyes. Hybridization images were collected using a laser scanner (GenePix 4000B, Molecular Device) and digitized using Array-Pro image analysis software (Media Cybernetics). Data were analyzed by first subtracting the background and normalizing the signals using a LOWESS filter (Locally-weighted Regression) [35]. For two-color experiments, the ratio of the two sets of detected signals (log_2_ transformed, balanced) and p-values of the t-tests were calculated; differentially detected signals were those with p-values less than 0.01. The normalized signal intensity from 8 samples (Week 4 of human embryonic development: ZN18, ZN46/47, ZN75; Week 5: ZN38, ZN43, ZN63-1; Week 6: ZN61, ZN70) was used the clustering analysis using a one-way analysis of variance (ANOVA) by a service provider (LC Sciences). Normalized data for all arrays have been deposited in the Gene Expression Omnibus (GEO) at the National Center for Biotechnology Information (NCBI), accessible through GEO Series accession number GSE46795 (http://www.ncbi.nlm.nih.gov/geo/query/acc.cgi?acc=GSE46795).

High-density multiple organ tumor and normal tissue microarrays (catalog MC5003), containing 18 tumor types (20 cases per type) and normal corresponding controls (5 cases per type), were purchased from US Biomax. The miRNA and scrambled oligonucleotides for *in situ* hybridization were purchased as digoxigenin-labeled locked nucleic acid (LNA) probes from Exiqon (Denmark). The sequence of the LNA detection probes were: LNA-638: 5’-DIG-AGGCCGCCACCCGCCCGCGATCCT-3’; LNA-720: 5’-DIG-TGGAGGCCCCAGCGAGA-3’; and LNA-1280: 5’-DIG-GGGTGGCAGCGGTGGGA-3’. LNA-U6: 5’-DIG-CACGAATTTGCGTGTCATCCTT-3’ and LNA-scramble: 5’-DIG-GTGTAACACGTCTATACGCCCA-3’ were used as positive and negative control probes, respectively. The *in situ* hybridization of tissue array for miRNAs experssion was performed using a service provider (Shaanxi Chaoying Biotechnology CO, LTD, Shaanxi China), and slides were read by two independent researchers. The intensity of the staining was scored as negative (-/0), weak (+/1), moderate (++/2), or strong (+++/3) as previously described [36,37].

### miRNA real-time polymerase chain reaction (PCR)

miRNA RT-PCR assay was performed using a service provider (LC Sciences). Briefly, miRNA quantifications were examined using TaqMan^®^ MicroRNA Assays and TaqMan® Universal PCR Master Mix and analyzed by ABI PRISM 7000 Sequence Detection System. RNU24 was used as an internal control to determine relative miRNA expression. Each sample was performed in triplicate.

### Statistical analyses

One-way analysis of variance (ANOVA) was performed using the normalized data for week 4–6 to identify miRNAs whose expression changed. To compare miRNAs expression in human normal and cancer tissues, two-tailed Student’s *t* tests were performed using the above scores of samples.

## Results and Discussion

### Characterization of miRNA expression profiles during human embryonic development

**Figure 1:**
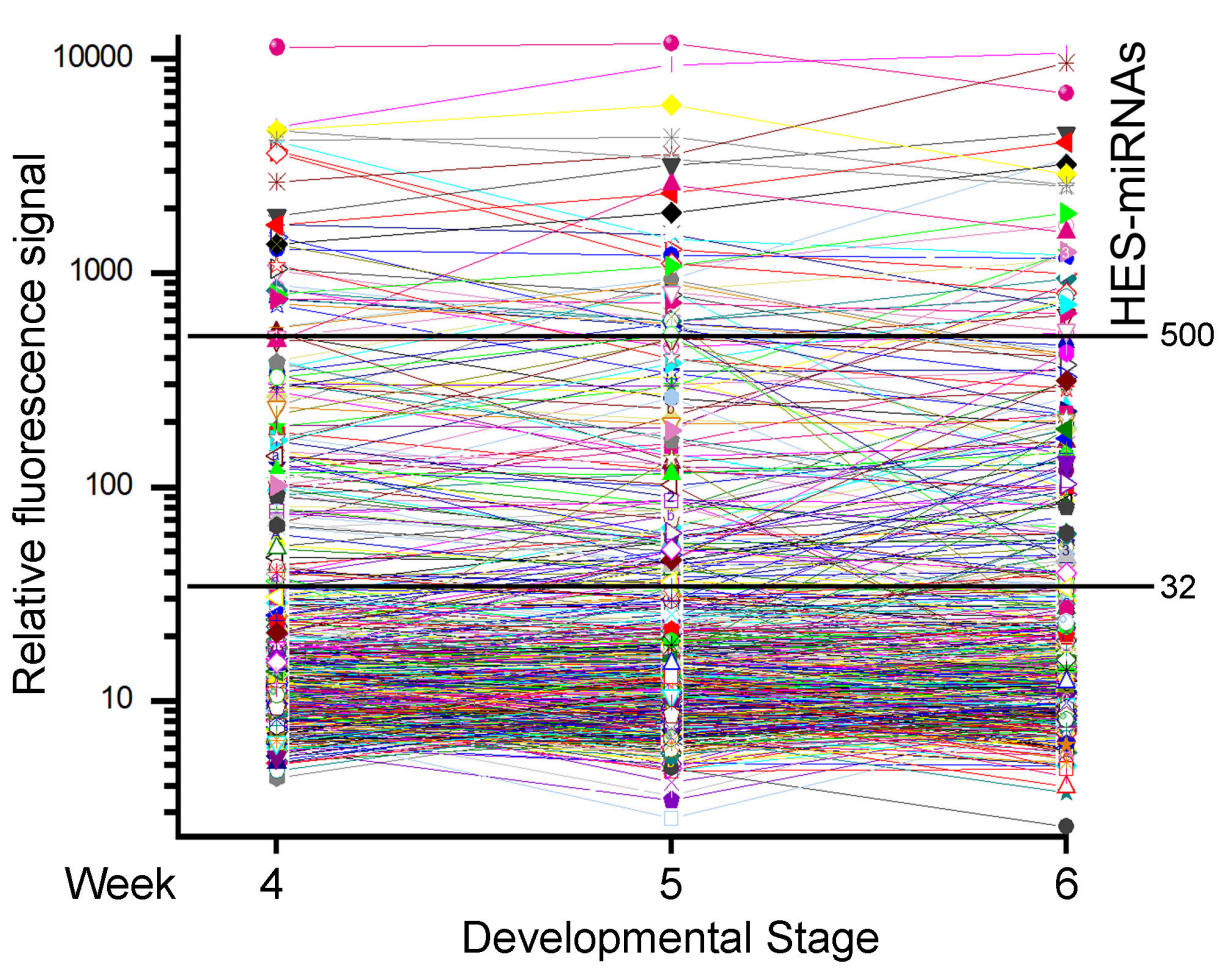
Expression overview of the 835 miRNAs during human embryonic development MiRNA expression levels during human embryonic development (weeks 4, 5, and 6 after fertilization) with signal strength greater 32 were considered as detected expression. A strong signal threshold of miRNA detection was defined as 500 after normalization. MiRNAs with a fluorescence signal greater than 500 at any stage at weeks 4, 5 or 6 were defined as HES-miRNAs.

To identify the miRNA expression profiles of human embryos, miRNA microarrays assays were performed in human embryo samples (weeks 4, 5, and 6 after fertilization) using µParaflo® technology, and the microarrays raw data were analyzed. The array covers all miRNA transcripts available in the Sanger miRBase database (release 10.1). For details, see the Experimental Procedures and Supplemental Data (Table S1 available online). Accordingly, miRNAs exhibiting signal strengths greater than 32 were considered as detected expression. After normalization, a strong signal threshold of miRNA detection was defined as 500 (Figure 1). We designated these 50 miRNAs (~6%), in which the expression signal was greater than 500 of any stage at weeks 4, 5, or 6 during human embryonic development, as human embryo-specific miRNAs (HES-miRNAs) (Table 1).

**Table 1:** List of human-embryo-specific-miRNAs.

miRNA	Week 4	Week 5	Week 6
hsa-miR-103	1020.97	589.63	775.30
hsa-miR-106a	3717.47	1283.56	988.08
hsa-miR-106b	705.36	336.58	217.08
hsa-miR-107	837.20	446.95	520.07
hsa-miR-10b	1679.97	1511.97	688.29
hsa-miR-122	4805.00	9396.85	10671.26
hsa-miR-1246	356.13	816.55	100.26
hsa-miR-125a-5p	387.96	827.09	1146.67
hsa-miR-125b	558.09	937.53	3407.28
hsa-miR-126	513.43	257.52	195.18
hsa-miR-1268	67.10	212.18	725.03
hsa-miR-1275	238.04	928.53	413.71
hsa-miR-1280	189.87	292.81	1238.08
hsa-miR-130a	522.76	130.33	161.38
hsa-miR-130b	827.85	593.00	936.95
hsa-miR-151-5p	713.59	579.19	423.27
hsa-miR-15b	1041.17	788.21	368.30
hsa-miR-16	1080.15	395.87	286.75
hsa-miR-17	4108.91	1437.68	1210.55
hsa-miR-181a	312.21	269.73	741.07
hsa-miR-181b	192.98	187.58	873.77
hsa-miR-182	341.78	579.71	453.59
hsa-miR-191	482.51	483.55	631.69
hsa-miR-199a-3p	1284.65	1201.39	1165.51
hsa-miR-19b	758.72	559.73	223.40
hsa-miR-206	791.12	1067.48	1658.21
hsa-miR-20a	3614.13	1105.53	804.23
hsa-miR-20b	1471.15	540.45	323.50
hsa-miR-214	2654.73	3566.04	9605.29
hsa-miR-23b	745.25	594.28	767.96
hsa-miR-25	1871.25	1553.32	1021.11
hsa-miR-26a	4645.23	3390.43	2536.21
hsa-miR-320a	1849.63	3185.42	4495.39
hsa-miR-320b	1358.11	1904.94	3209.74
hsa-miR-320c	1666.09	2358.80	4068.96
hsa-miR-320d	790.28	1072.82	1897.73
hsa-miR-361-5p	751.92	719.17	643.41
hsa-miR-423-5p	553.06	888.43	400.99
hsa-miR-432	499.71	807.49	534.52
hsa-miR-451	1355.12	622.20	18.66
hsa-miR-483-5p	480.51	2580.41	1537.07
hsa-miR-574-5p	163.53	375.89	709.17
hsa-miR-638	4622.85	6092.42	2891.58
hsa-miR-663	290.15	525.92	44.41
hsa-miR-720	101.92	181.66	1255.10
hsa-miR-92a	11338.08	11838.01	6909.45
hsa-miR-92b	4171.40	4315.19	2556.64
hsa-miR-93	872.20	552.87	445.45
hsa-miR-936	323.52	512.72	35.61
hsa-miR-99b	510.57	635.73	1232.78

After normalization, a strong signal threshold of miRNA detection was defined as 500 (Figure 1), and 50 miRNAs (~6%, 50/835), in which the expression signal was greater than 500 of any stage at weeks 4, 5, or 6 during human embryonic development, were designated as human-embryo-specific miRNAs (HES-miRNAs).

**Figure 2:**
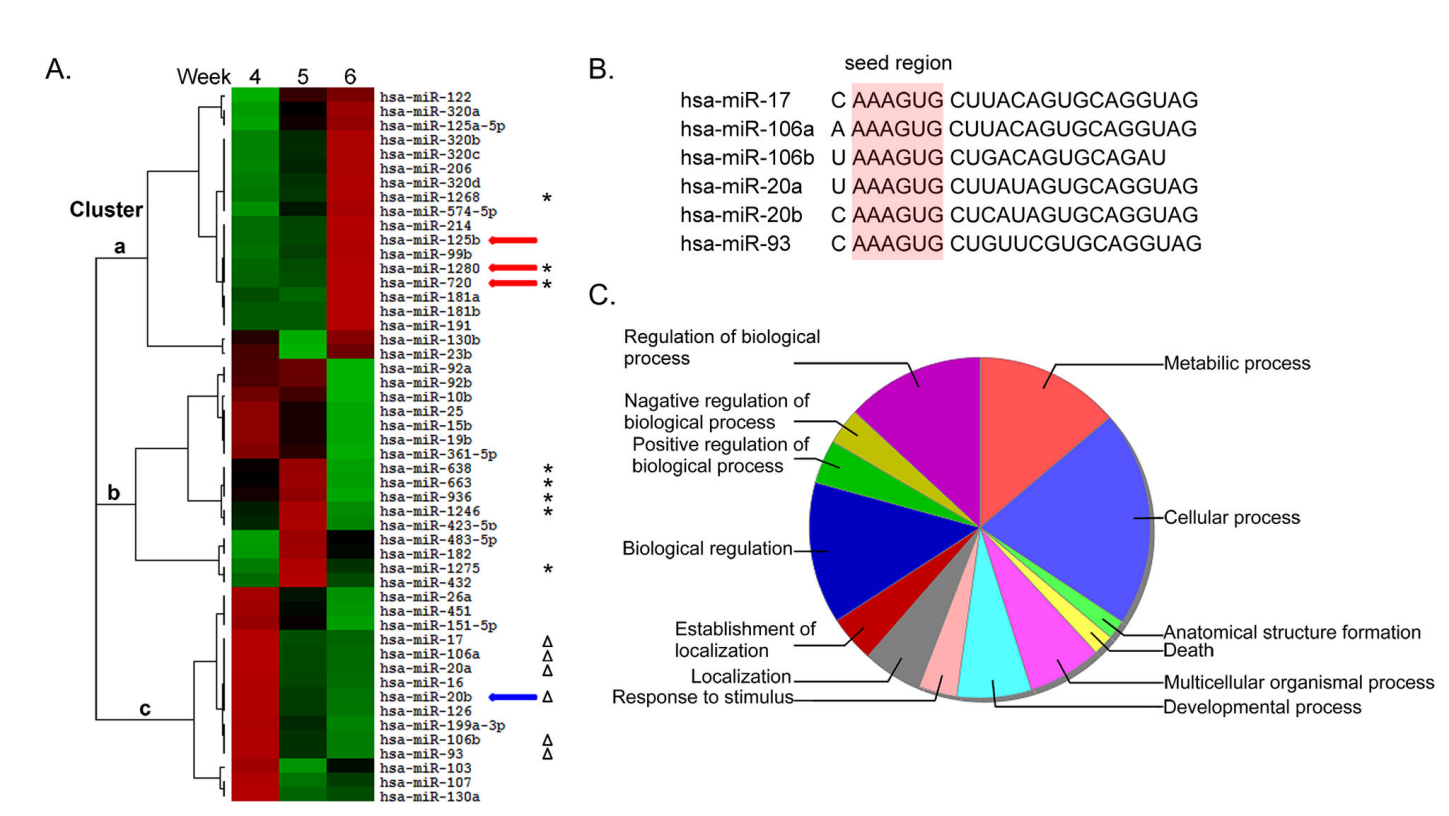
Hierarchical clustering analyses of the expression of 50 HES-miRNAs (A) Fifty HES-miRNAs were divided into 3 clusters: clusters a, b, and c. The arrow shows the miRNAs that were selected to further validate the microarray data using microRNA qRT-PCR (Figure 3). The asterisk shows the non-conserved or primate-specific HES-miRNAs (Figure S4). The hollow triangle indicates miRNAs harborring the same seed region sequence among cluster c. (B) Figure 2B shows the mature sequence of six miRNAs (miR-17, -106a, -106b, -20a, -20b, -93) with the identical seed region tagged with light red shadow. (C) Pie chart shows functional pattern analysis (Gene Ontology) of the conserved targets (1245 transcripts), predicted by TargetScan (http://www.targetscan.org/vert_60/), of the six miRNAs.

The expression profiles show that the expression of most miRNAs is low during early human embryonic development, in which approximately 80% of miRNAs (666 of 835 miRNAs) exhibiting signal strength were considered as undetected expression (Figure 1 and Table S1). These data are similar to the findings that most miRNAs were not detected during early zebrafish development [10]. Hierarchical cluster assays of HES-miRNA expression (Figure 2A) and 169 miRNAs exhibiting signal strength over 32 (Figure S1) during human embryonic development are shown. In Figure 2A, the following three clusters of HES-miRNAs expressions were identified: Cluster a, upregulated at week 6; Cluster b, downregulated at week 6; and Cluster c, upregulated at week 4. In Figure S1, four clusters of 169 miRNAs were identified: clusters 1, 2, 3, and 4. Cluster a miRNAs in Figure 2A increased during the three human embryonic stages (week 6 > week 5 > week 4), and included some miRNA species reported to be enriched in various developmental and disease processes (e.g., hsa-miR-122, -206, -214, -181 family, and -125 family) [38]. Cluster c miRNAs that decreased during the three human embryonic stages (week 4 > week 5 > week 6) included some miRNAs that were also enriched in various developmental and disease processes (e.g., hsa-miR-451, -106, -16, and -17) (Figure 2A) [38]. However, the expression of many cluster b members that increased from week 4 to week 5, but decreased from week 5 to week 6, have unknown functions in development and disease processes.

Among the HES-miRNAs (Table 1 and Figure 2A), some have been experimentally confirmed to play pivotal roles in differentiation and disease. For instance, the mammalian liver-specific miRNA, miR-122, which had the highest expression level in week 6 of human embryonic development, is frequently suppressed in primary hepatocellular carcinomas [39,40], and is also essential for hepatitis C virus RNA accumulation in cultured liver cells [10,41–47]. Hsa-miR-214 is related to muscle development and bone formation, and is involved in gastric and ovarian cancer [48–53]. Hsa-miR-92a/b, as members of the miR-17~92 family, is involved in many solid tumors and leukemia [54–57]. Hsa-miR-106a is involved in regulation of induced pluripotent stem cell generation, posttranscriptional regulation of interleukin-10 expression, gastric cancer, and astrocytoma [58–61]. Therefore, our results strongly support the notion that there is striking similarity between early embryonic development and tumorigenesis [22].

Moreover, these highly expressed miRNAs, as well as those that have low expression, may play different regulatory roles in different stages of human embryonic development. Alternatively, these miRNAs with high expression levels have strong tissue specificity, such as hsa-miR-122.

Among cluster c, it’s amazing that we noticed 6 miRNAs (hsa-miR-17, -106a, -106b, -20a, -20b and -93), which contain the same seed region sequcence AAAGUG (Figure 2B), were downregulated at week 6 of human embryonic development, and might play the similar biological functions in human embryogenesis. The Hannon and Hammond laboratories provided direct experimental results that these microRNAs, encoded by the miR-17-92 cluster and its paralogs (the miR-106a-363 cluster and miR-106b-25 clusters), have oncogenic activity in solid tumor [62]. Ventura and colleagues documented strong evidences that deletion of the miR-17-92 cluster resulted in severely hypoplastic lungs and reduction in pre-B cells numbers in mice, finally leading to smaller embryos even immediate postnatal death [63]. Considering the vital role of these miRNAs in development and tumorigenesis, we predicted all targets of these 6 miRNAs by TargetScan, and then the conserved targets (1245 transcripts) were further analyzed. During early stage of human embryonic development, one important event associated with the developmental transition from the early cell proliferation phase to organogenesis and histogenesis is that the number of stem cells or undifferentiated cells is reduced because more cells begin to differentiate into different organ/tissue-specific cell types. During early stage of human embryonic development, one important event associated with the developmental transition from the early cell proliferation phase to organogenesis and histogenesis is presented. Functional pattern analysis (Gene Ontology) from the conserved target (1245 transcripts) of 6 miRNAs shows that the vast majority of these genes in the GO categories related to biological regulation, regulation of biological process, metabilic process, cellular process, multicellular organismal process and developmental process (Figure 2C). Recently reported some targets of miR-17~92 family, such as CDKN1A (p21) [64], BIM [65], RUNX1 [66], both of them are included in these GO categories, which suggest that these genes are irreplaceable position in controlling of cell cycle, cell apopotosis, and differentiation of hematopoietic progenitor cells. And these results could help us better understand the role of HES-miRNAs in human embryogenesis and tumorigenesis.

**Figure 3:**
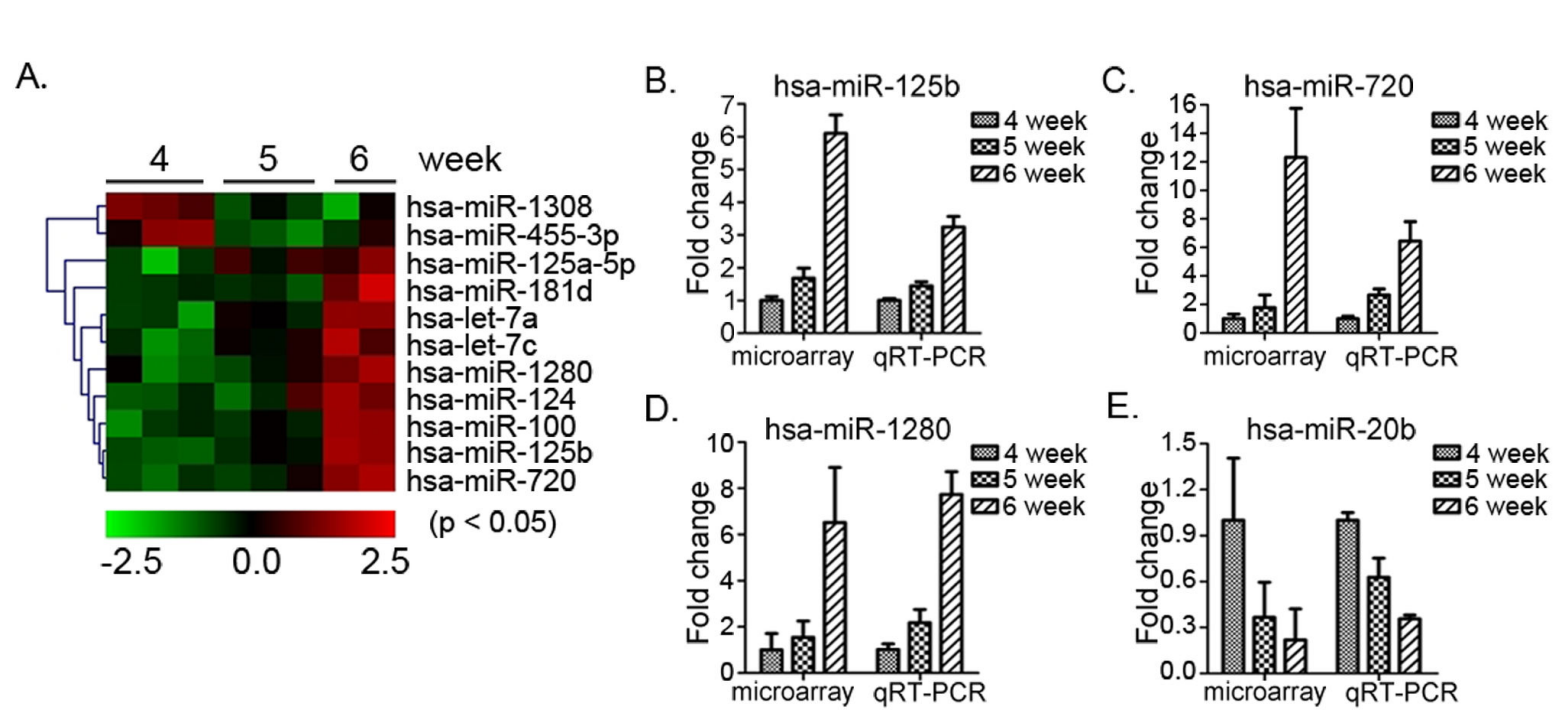
miRNA qRT-PCR analyses validated the miRNA microarray results Four miRNAs [hsa-miR-125b (B), hsa-miR-720 (C), hsa-miR-1280 (D), and hsa-miR-20b (E)], which were differentially expressed (p<0.05, Figure 3A) and (p<0.10, Figure S2) during weeks 4, 5, and 6 of human embryonic development, were chosen. qRT-PCR was performed and U6 snRNA was used as an internal control to determine the relative miRNA expression.

In addition, to validate the quality of the miRNA microarray data, four miRNAs (hsa-miR-125b, -720, -1280, and -20b, Figure 3 and Figure S2) whose expression levels changed significantly during development were validated using TaqMan® real-time RT-PCR miRNA assays. The trends of miRNA expression were consistent with that observed in the miRNA microarray data (Figure 3B, C, D, and E).

### Human-Mouse Comparative Analyses of miRNA Expression During Embryonic Development

To compare the miRNA expression profiles between human and mouse embryonic development, we conducted a comparative analysis relative to previously published microRNA expression profiles in mouse embryos covering prenatal development (E9.5, E10.5, and E11.5) [8], which correspond with weeks 4, 5, and 6 of human embryonic development [67,68]. Given the identical miRNAs in human and mouse, we constructed an intersection analysis between 835 human miRNAs in this study and 390 mouse miRNAs, and identified 195 homologous miRNAs (Table S2). The hierarchical clustering assays of human-mouse homology miRNAs are shown in Figure S3A and B. Venn diagrams (Figures S3C and S3D) showed that 38 or 32 human-mouse homology miRNAs were upregulated or downregulated, respectively, during human and mouse embryonic development. Among these miRNAs, let-7 family members, miR-34a, miR-221/222, miR-145, miR-125b, miR-15a, and miR-223 have been proven to play multifarious roles during development and pathogenesis in various mammalian organs [38]. These results further suggest that some homologous human-mouse miRNAs are important for development and pathogenesis, whereas those that are not homologous are likely involved in different development processes that reflect the developmental divergence between human and mouse.

### Conservative properties of HES-miRNAs

A previous study showed that many known miRNA genes have a typical conservation pattern, persistent throughout the clusters, suggesting evolutionary and functional implications [69]. However, a substantial portion of microRNAs are confirmed as non-conserved or primate-specific miRNAs because many new human miRNAs have been continuously identified [70]. To define the conservation of HES-miRNAs, we used the miRviewer [71], which shows conservation of miRNA genes grouped by name. As shown in Figure S4, most HES-miRNAs are highly conserved, and their functions are well known. Remarkably, among HES-miRNAs, eight miRNAs, hsa-miR-638, -663, -720, -936, -1246, -1268, -1275, and -1280, are extremely non-conserved, even though most are primate-specific (Figure S4) [70,72]. Noticeably, the peak expression values of 5 of these miRNAs, miR-638, -663, -936, -1246, and -1275, are at 5 weeks of human embryonic development. In other words, these miRNAs may function only at a specific human embryonic developmental stage. Furthermore, we know very little about the biological functions of these miRNAs in mammalian organogenesis and pathogenesis. However, because the expression of these non-conserved or primate-specific miRNAs is high during human embryonic development, these miRNAs likely play pivotal roles in mammalian organogenesis and/or pathogenesis. In fact, a few studies indicate that these non-conserved or primate-specific HES-miRNAs may be associated with all kinds of disease [73–82]. The function of these non-conserved or primate-specific HES-miRNAs remains to be determined.

### Comparative analyses of the expression of hsa-miR-638, -720, and -1280 in normal and malignant tissues

Many studies have indicated that miRNAs are involved in diverse aspects of animal developmental processes and disease [4,16–21,38,83–89]. To further validate the relationship between the HES-miRNAs and various solid malignant tumors, we focus on three non-conserved or primate-specific HES-miRNAs, hsa-miR-638, -720, and -1280 (Table 1), of highly expressed or exhibit expression changes during week 4-6 of human embryonic development for further functional investigation. We examined whether the expression of the three HES-miRNAs differs significantly between various human tumors and the corresponding normal tissue.

**Figure 4:**
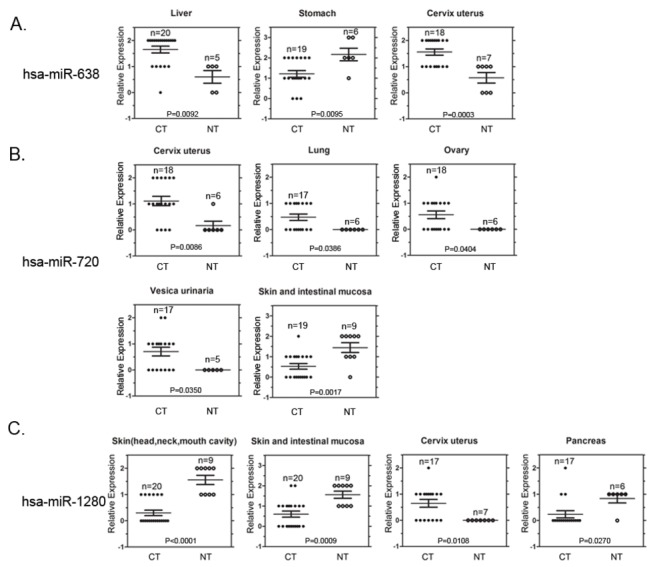
Statistical analyses of three HES-miRNAs (hsa-miR-638, -720, and -1280) expression using *in situ* hybridization High-density multiple organ tumor and normal tissue microarrays containing 500 tissue-dots with 18 tumor types and normal corresponding control tissues. Digoxigenin-labeled locked nucleic acid (LNA) probes (LNA-638, LNA-720, and LNA-1280) were used to specifically detect the miRNA expression on the tissue chip. (A) Aberrant expression of hsa-miR-638 on tissue microarray. Significant up-regulation of miR-638 can be observed in hepatocellular liver cancer and cervix uteri squamous cell carcinoma. miR-638 down-regulation is found in stomach adenocarcinoma versus the corresponding normal tissues (p=0.0092, p=0.0003, and p=0.0095, respectively) (B) Aberrant expression of hsa-miR-720 on tissue microarray. Hsa-miR-720 is significantly upregulated in cervix uteri squamous cell carcinoma, lung squamous cell adenocarcinoma, ovary adenocarcinoma, and urothelial carcinoma versus the corresponding normal tissues (p=0.0086, p=0.0386, p=0.0404, and p=0.035, respectively). Hsa-miR-720 is significantly downregulated in intestinal mucosa malignant tissues versus normal skin tissues (p=0.0017). (C) Aberrant expression of hsa-miR-1280 on tissue microarray. Hsa-miR-1280 is downregulated in squamous cell carcinoma, intestinal mucosa malignant tissue, and pancreatic adenocarcinoma versus the corresponding normal tissues (p<0.0001, p=0.0009, and p=0.0270, respectively). Hsa-miR-1280 is upregulated significantly in cervix uteri squamous cell carcinoma tissues versus normal cervix uteri tissues (p=0.01076). MiRNA expression levels (y-axis) with a score=0, 1, 2, or 3, indicate negative, weak, medium, or strong staining intensity, respectively. n indicates the numbers of the specimens studied. NT indicates normal tissue samples; CT indicates carcinoma tissue samples. Two-tailed student *t* tests were performed to compare miRNA expression in normal and cancerous tissues.

**Figure 5:**
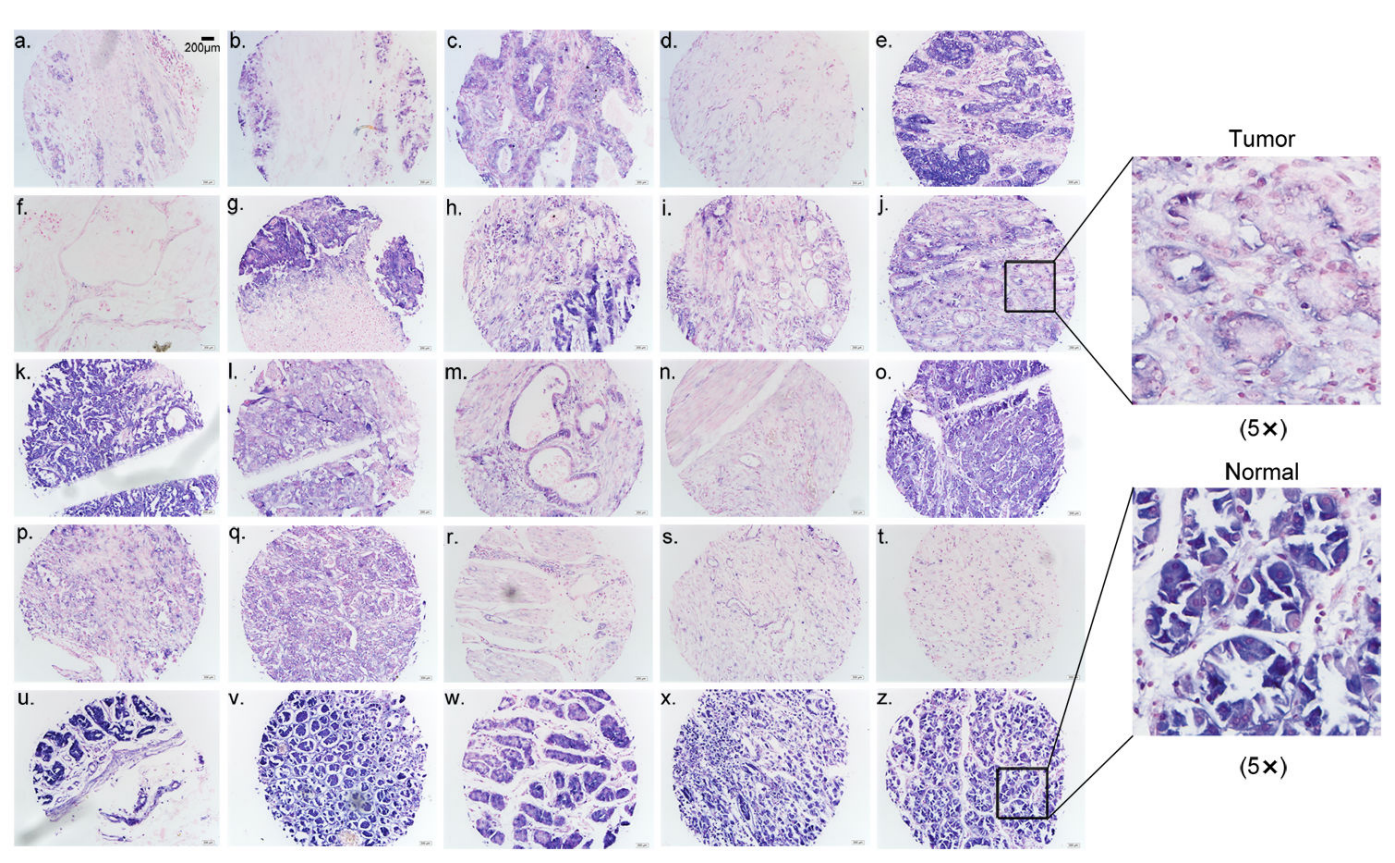
hsa-miR-638 expression in stomach adenocarcinoma and corresponding normal tissues. Panels a through t show stomach adenocarcinoma samples and panels u through z show normal stomach tissue. The blue staining signal indicates expression of hsa-miR-638. The red staining shows the cell nucleus (scale 200μm as Figure 5a). j2 shows higher magnification (5x) of the indicated area in figure 5j, and z2 shows higher magnification (5x) of the indicated area in figure 5z.

Using the tissue microarray MC5003 and highly specific and sensitive LNA-modified oligonucleotide probes, the expression profiles of the 3 miRNAs in normal and cancerous tissues were detected using *in situ* hybridization. U6 snoRNA and scramble-miR probes were used as positive and negative control probes, respectively. The statistical results show that the expression of hsa-miR-638 is significantly upregulated in hepatocellular liver cancer tissues (n=20) versus normal liver tissues (n=5) (p=0.0092), and in cervix uteri squamous cell carcinoma tissues (n=18) versus normal cervix uteri tissues (n=7) (p=0.0003). In contrast, the expression of hsa-miR-638 was significantly downregulated in stomach adenocarcinoma tissues (n=19) versus normal stomach tissues (n=6) (p=0.0095) (Figure 4A). These data agree with the results from Tsukamoto et al. showing downregulation of this miRNA in gastric cancer [90]. hsa-miR-720 expression is significantly upregulated in cervix uteri squamous cell carcinoma tissues (n=18) versus normal cervix uteri tissues (n=6) (p=0.0086), in lung squamous cell carcinoma/adenocarcinoma tissues (n=17) versus normal lung tissues (n=6) (p=0.0386), in ovary cystadenocarcinoma/adenocarcinoma tissues (n=18) versus normal ovary tissue (n=6) (p=0.04045), and in urothelial carcinoma tissues (n=17) versus normal vesica urinaria tissues (n=5) (p=0.03504) (Figure 4B). In addition, hsa-miR-720 expression is significantly downregulated in intestinal mucosa malignant tissues (n=19) versus normal skin tissues (n=9) (p=0.0017) (Figure 4B). The expression of hsa-miR-1280 is significantly downregulated in squamous cell carcinoma tissues of head and neck skin (n=20) versus normal skin tissues (n=9) (p<0.0001), in intestinal mucosa malignant tissues (n=20) versus normal skin tissues (n=9) (p=0.0009), and in pancreatic adenocarcinoma tissues (n=17) versus normal pancreatic tissues (n=6) (p=0.0270) (Figure 4C). In contrast, hsa-miR-1280 expression is significantly upregulated in cervix uteri squamous cell carcinoma tissues (n=17) versus normal cervix uteri tissues (n=7) (p=0.01076) (Figure 4C). Representative examples of three miRNAs that were differentially expressed in tumor tissues versus normal tissues are shown in Figures 5, S5, S6, and S7. The results suggest that these HES-miRNAs are closely related to the pathological processes of the various tumors.

In summary, our studies herein reveal that the expression levels of most miRNAs during human embryogenesis are very low. We designated 50 (~6%) of the 835 miRNAs regulated in weeks 4 through 6 of human embryonic development as HES-miRNAs, and demonstrated that some non-conserved or primate-specific HES-miRNAs are involved in tumorigenesis, thus supporting the hypothesis that early embryonic development shares many similarities with cancer development in biological behavior as well as molecularly [22]. However, the functions of the non-conserved or primate-specific HES-miRNAs remain to be experimentally established, which will help us further understand the complicated molecular modulation network that occurs during human embryonic development.

## Supporting Information

Figure S1 **Hierarchical clustering analyses of the expression of 169 miRNAs exhibiting signal strengths greater than 32 miRNAs (n=169) were divided into 4 clusters: clusters 1, 2, 3, and 4.** The arrow shows the miRNAs that were selected be validated by microRNA qRT-PCR (Figure 3). The asterisk shows the non-conserved or primate-specific HES-miRNAs (Figure S4).
(TIF)

Figure S2 **Clustering analyses of miRNA expression (*p*<0.10) during human embryonic development Red and green indicate high and low expression levels, respectively.**
(TIF)

Figure S3 **Conserved miRNA expression patterns and biological characteristics of human-mouse homologs during embryonic development.** Unsupervised hierarchical clustering analyses of miRNA expression proﬁles in humans (weeks 4, 5, and 6, Figure S3A) and mouse embryo samples (E9.5, E10.5, and E11.5, Figure S3B). The mouse embryo microarray data were published in 2006 by Mineno and colleagues. The color in each lattice reﬂects the expression level of the miRNA in the corresponding sample. The increasing intensities of red indicate that a speciﬁc miRNA has a higher expression in the given sample. The increasing intensities of green indicate that this miRNA has lower expression. Venn diagrams depict the intersection miRNAs between humans (weeks 4, 5, and 6) and mouse embryo samples (E9.5, E10.5, and E11.5) and show that 38 and 32 human-mouse homology miRNAs were upregulated (C) and downregulated (D), respectively, during human and mouse embryonic development.
(TIF)

Figure S4 **Conservation analyses of the HES-miRNAs.** The miRviewer shows conservation of the HES-miRNA genes, grouped by name. The miRNAs that only can be discovered in human or other primates, including miR-638, -663, -720, -936, -1246, -1268, -1275, and -1280, were separated below. The increasing intensities of green indicate that a speciﬁc miRNA (or miRNA family) has higher conservation in the given species. Numbers in brackets indicate the numbers of the miRNAs in a given miRNA family. Most share similar conservation results.
(TIF)

Figure S5 **hsa-miR-638 expression in cervix uteri adenocarcinoma and corresponding normal tissues.** Panels a through t show cervix uteri adenocarcinoma samples and panels u through z show normal cervix uteri tissues. The blue staining signal indicates expression of hsa-miR-638. The red staining shows the cell nucleus (scale 200µm as Figure S5a).
(TIF)

Figure S6 **hsa-miR-720 expression in cervix uteri adenocarcinoma and corresponding normal tissues.** Panels a through t show cervix uteri adenocarcinoma samples and panels u through z show normal cervix uteri tissues. The blue staining signal indicates expression of hsa-miR-720. The red staining shows the cell nucleus (scale 200µm as Figure S6a).
(TIF)

Figure S7 **hsa-miR-1280 expression in skin tumors and corresponding normal tissues.** Panels a through t show skin tumor samples and panels u through z show normal skin tissues. The blue staining signal indicates expression of hsa-miR-1280. The red staining shows the cell nucleus (scale 200µm as Figure S7a).
(TIF)

Table S1 **The expressions of 835 miRNAs during week 4-6 of human embryonic development.**
(XLS)

Table S2 **List of 195 homologous human and mouse miRNAs.**
(XLS)
