# Supplementary figures and images for: Characterization of MicroRNA Expression Profiles and the Discovery of Novel MicroRNAs Involved in Cancer during Human Embryonic Development
Source: PLoS One. 2013 Aug 2;8(8):e69230. doi: 10.1371/journal.pone.0069230 (PMC3732277; doi:10.1371/journal.pone.0069230)

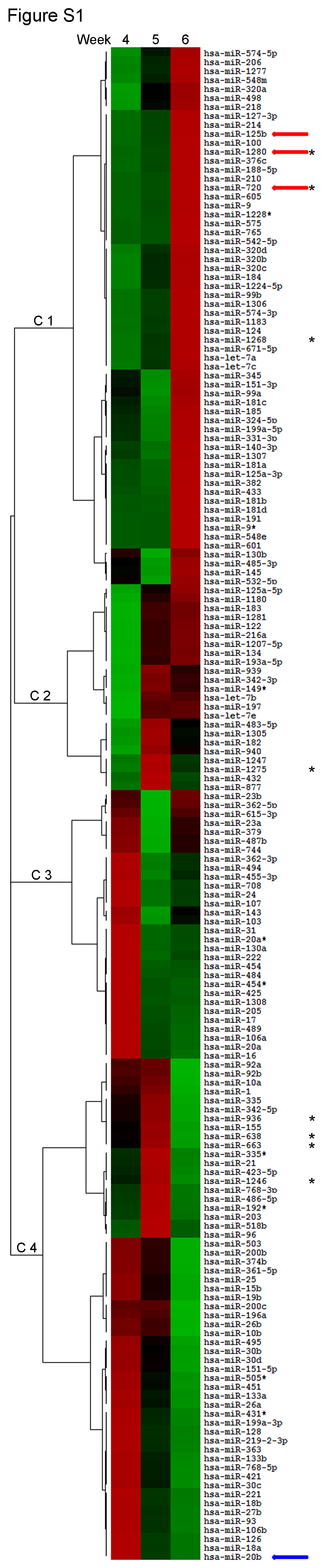

Supplement: Figure S1 — Hierarchical clustering analyses of the expression of 169 miRNAs exhibiting signal strengths greater than 32 miRNAs (n=169) were divided into 4 clusters: clusters 1, 2, 3, and 4. The arrow shows the miRNAs that were selected be validated by microRNA qRT-PCR (Figure 3). The asterisk shows the non-conserved or primate-specific HES-miRNAs (Figure S4). (TIF) [file pone.0069230.s001.tif]

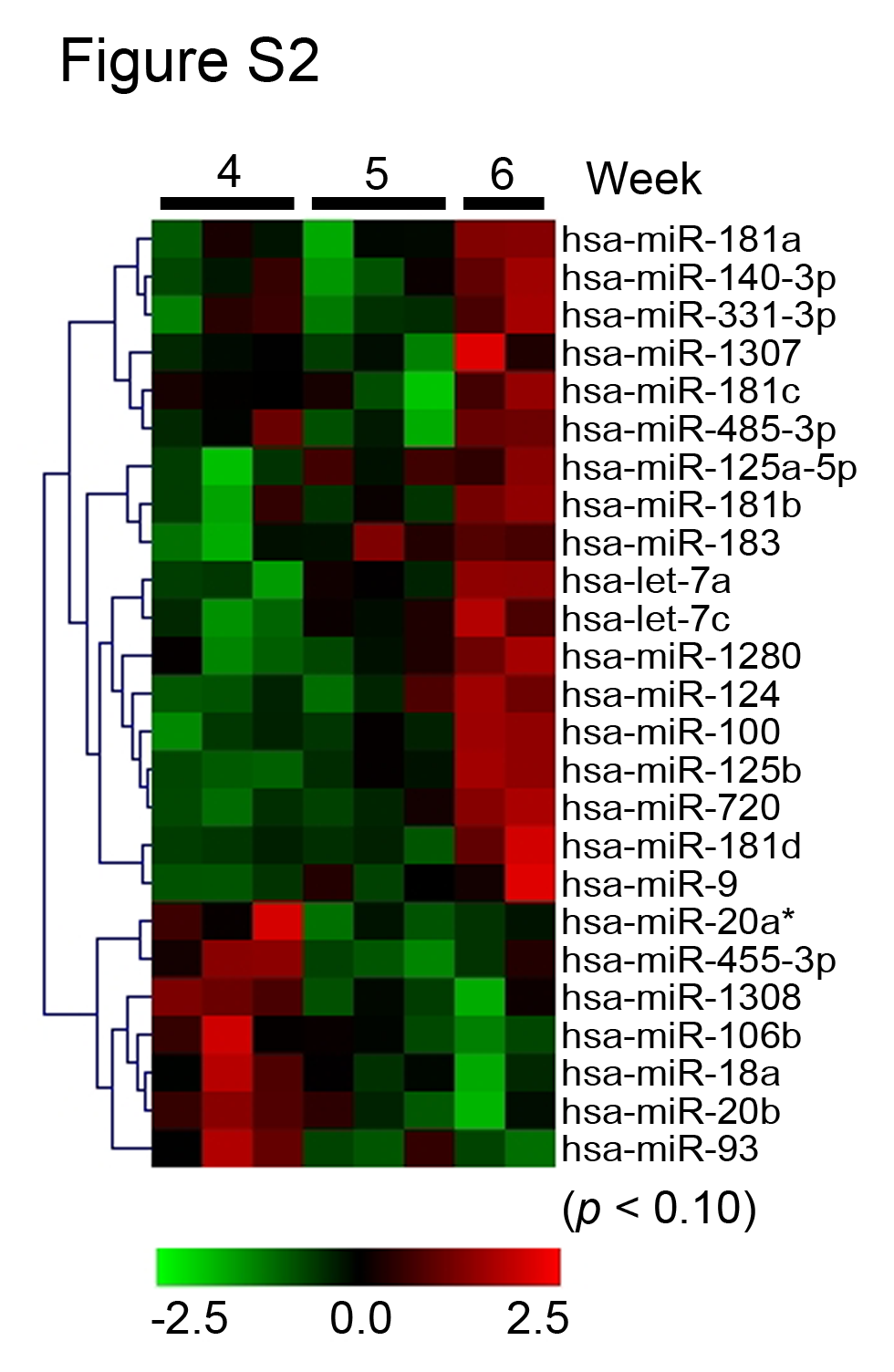

Supplement: Figure S2 — Clustering analyses of miRNA expression (p<0.10) during human embryonic development Red and green indicate high and low expression levels, respectively. (TIF) [file pone.0069230.s002.tif]

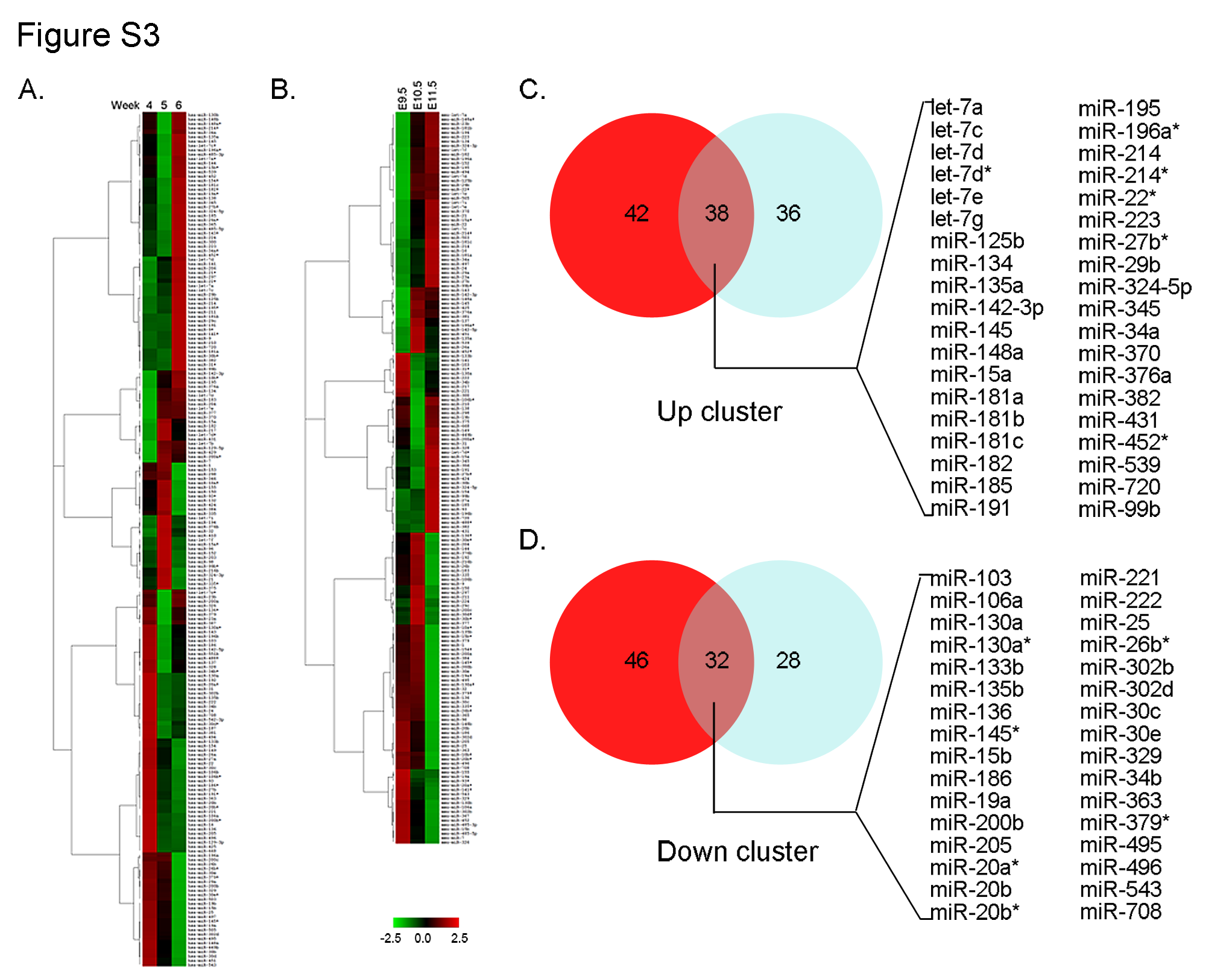

Supplement: Figure S3 — Conserved miRNA expression patterns and biological characteristics of human-mouse homologs during embryonic development. Unsupervised hierarchical clustering analyses of miRNA expression proﬁles in humans (weeks 4, 5, and 6, Figure S3A) and mouse embryo samples (E9.5, E10.5, and E11.5, Figure S3B). The mouse embryo microarray data were published in 2006 by Mineno and colleagues. The color in each lattice reﬂects the expression level of the miRNA in the corresponding sample. The increasing intensities of red indicate that a speciﬁc miRNA has a higher expression in the given sample. The increasing intensities of green indicate that this miRNA has lower expression. Venn diagrams depict the intersection miRNAs between humans (weeks 4, 5, and 6) and mouse embryo samples (E9.5, E10.5, and E11.5) and show that 38 and 32 human-mouse homology miRNAs were upregulated (C) and downregulated (D), respectively, during human and mouse embryonic development. (TIF) [file pone.0069230.s003.tif]

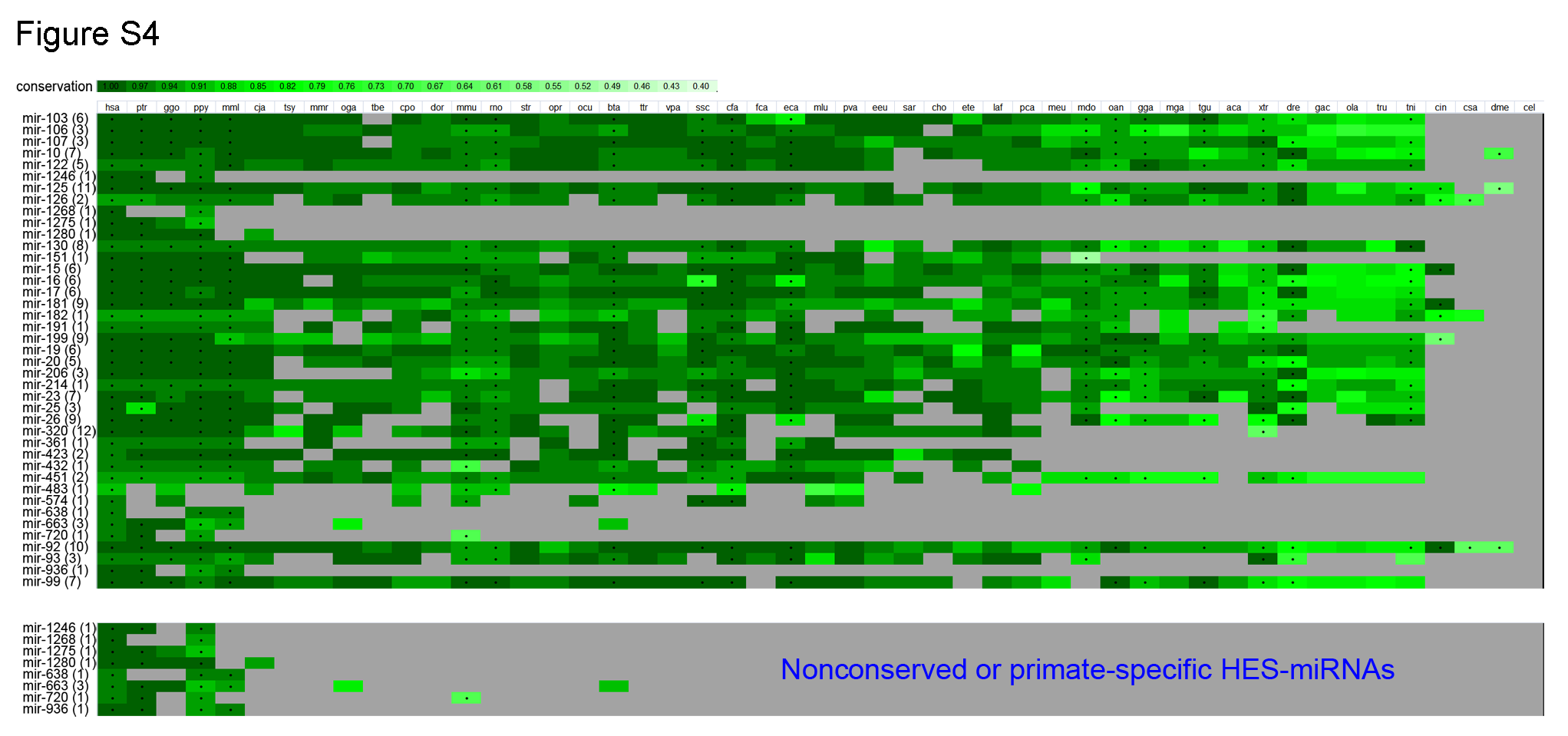

Supplement: Figure S4 — Conservation analyses of the HES-miRNAs. The miRviewer shows conservation of the HES-miRNA genes, grouped by name. The miRNAs that only can be discovered in human or other primates, including miR-638, -663, -720, -936, -1246, -1268, -1275, and -1280, were separated below. The increasing intensities of green indicate that a speciﬁc miRNA (or miRNA family) has higher conservation in the given species. Numbers in brackets indicate the numbers of the miRNAs in a given miRNA family. Most share similar conservation results. (TIF) [file pone.0069230.s004.tif]

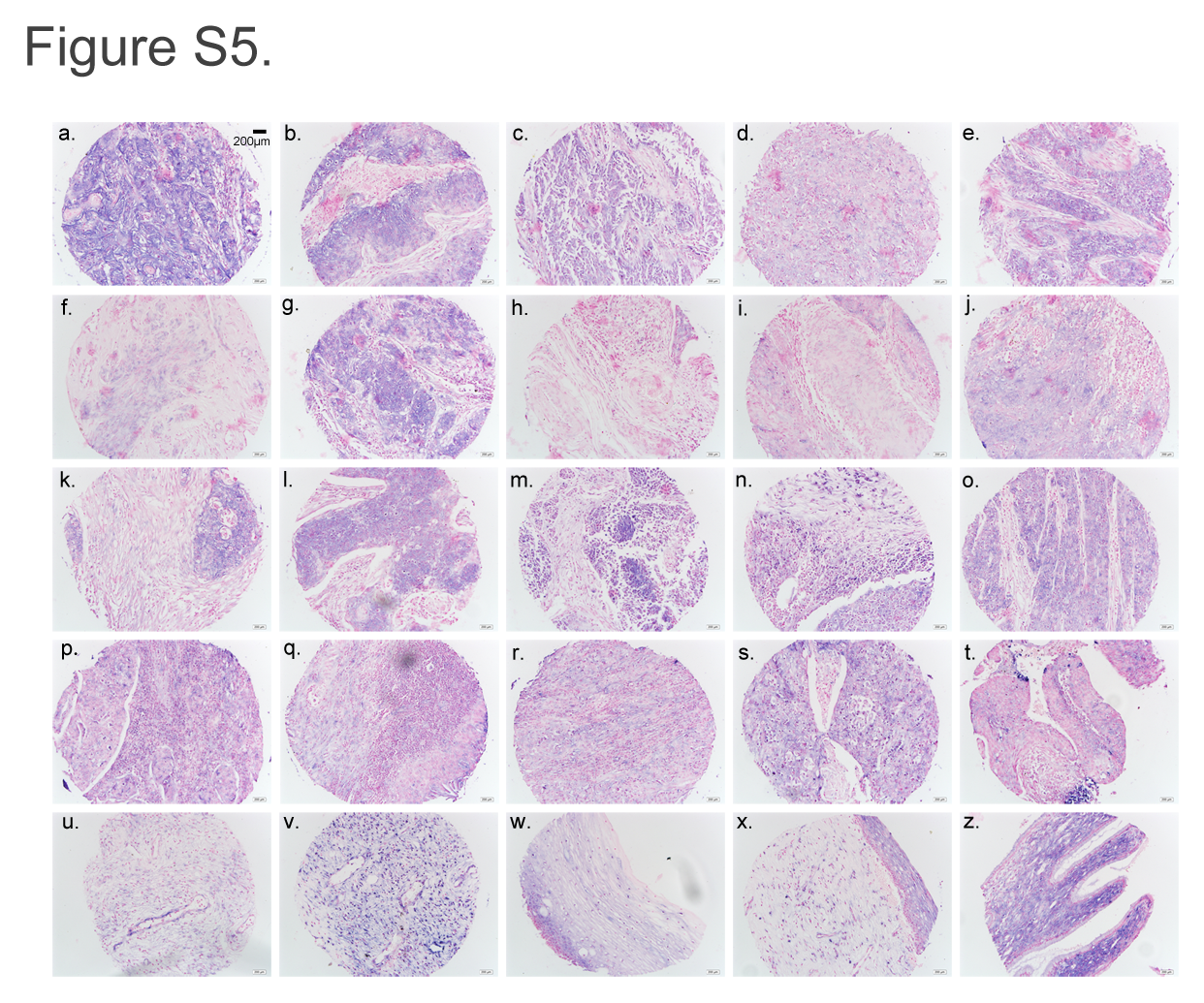

Supplement: Figure S5 — hsa-miR-638 expression in cervix uteri adenocarcinoma and corresponding normal tissues. Panels a through t show cervix uteri adenocarcinoma samples and panels u through z show normal cervix uteri tissues. The blue staining signal indicates expression of hsa-miR-638. The red staining shows the cell nucleus (scale 200µm as Figure S5a). (TIF) [file pone.0069230.s005.tif]

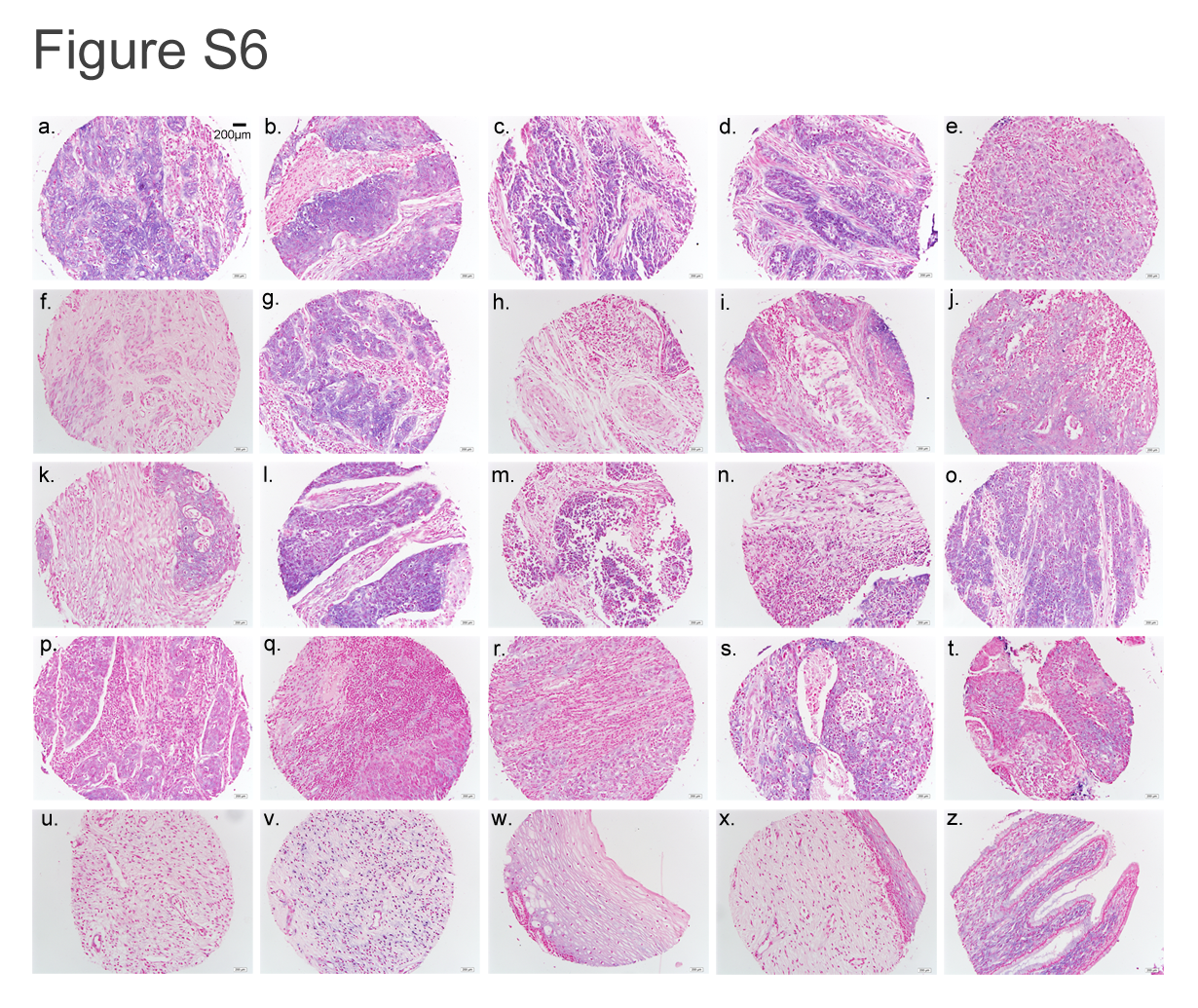

Supplement: Figure S6 — hsa-miR-720 expression in cervix uteri adenocarcinoma and corresponding normal tissues. Panels a through t show cervix uteri adenocarcinoma samples and panels u through z show normal cervix uteri tissues. The blue staining signal indicates expression of hsa-miR-720. The red staining shows the cell nucleus (scale 200µm as Figure S6a). (TIF) [file pone.0069230.s006.tif]

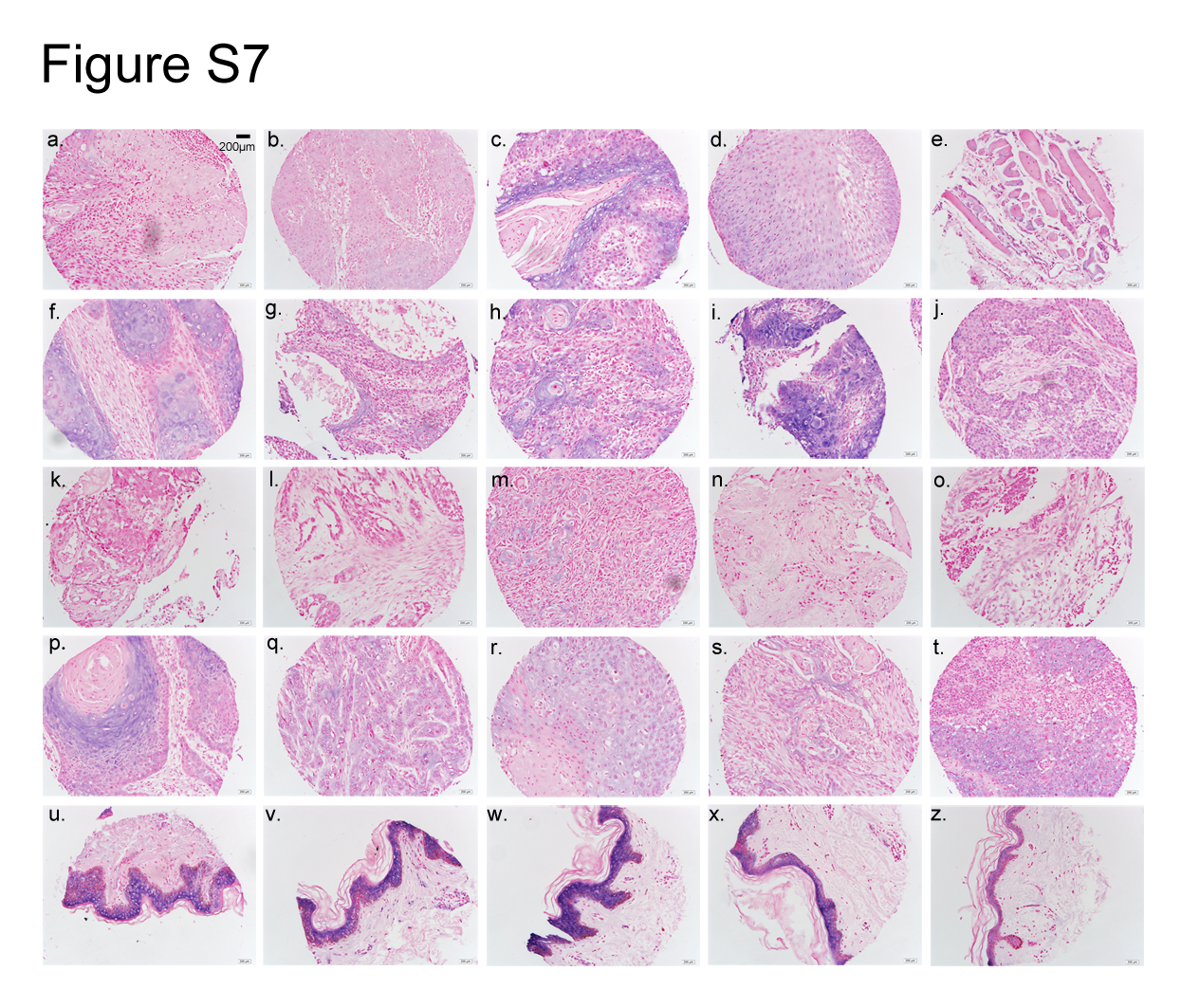

Supplement: Figure S7 — hsa-miR-1280 expression in skin tumors and corresponding normal tissues. Panels a through t show skin tumor samples and panels u through z show normal skin tissues. The blue staining signal indicates expression of hsa-miR-1280. The red staining shows the cell nucleus (scale 200µm as Figure S7a). (TIF) [file pone.0069230.s007.tif]
